# Enhancing Cryopreserved Sperm Quality in Chinese Rare Minnow *Gobiocypris rarus*: The Impact of Antifreeze Proteins

**DOI:** 10.3390/ijms251910364

**Published:** 2024-09-26

**Authors:** Huan Ye, Xin Li, Li Shen, Hao Du, Qing Zhang, Yongfeng He, Jinming Wu

**Affiliations:** 1 Key Laboratory of Freshwater Biodiversity Conservation, Ministry of Agriculture and Rural Affairs, Yangtze River Fisheries Research Institute, Chinese Academy of Fishery Sciences, Wuhan 430223, China; yehuan@yfi.ac.cn (H.Y.); lixin5993@126.com (X.L.); shenli@yfi.ac.cn (L.S.); duhao@yfi.ac.cn (H.D.); zq15797871593@126.com (Q.Z.); 2 Institute of Hydrobiology, Chinese Academy of Sciences, Wuhan 430072, China

**Keywords:** *Gobiocypris rarus*, antifreeze protein, cryopreservation, sperm quality, flow cytometer

## Abstract

The Chinese rare minnow (*Gobiocypris rarus*), an important model fish in China, faces endangerment in the wild. Sperm cryopreservation facilitates the development of new strains and germplasm conservation, but the quality of its cryopreserved sperm remains low. This study evaluates the protective effects of different concentrations of antifreeze proteins (AFP I and AFP III) on the cryopreservation of Chinese rare minnow sperm. Cryopreserved sperm showed significant declines in progressive motility, curvilinear velocity (VCL), average path velocity (VAP), and lifespan compared to fresh sperm, except for straight-line velocity (VSL). The cryomedium containing 10 μg/mL AFP I improved these parameters to their highest levels. However, no significant difference was found in progressive motility and kinetic parameters between cryopreserved sperm with and without AFPs. Cryopreserved sperm with 10 μg/mL AFP I showed the highest plasma membrane integrity, mitochondrial activity, and DNA integrity, significantly better than without AFPs; importantly, the fertilization rate of cryopreserved sperm with 10 μg/mL AFP I was not significantly different from that of fresh sperm. These results indicate that the addition of 10 μg/mL AFP I to the cryomedium for Chinese rare minnow sperm does not improve kinetic parameters but significantly enhances sperm quality, aiding in its new strain development and germplasm conservation.

## 1. Introduction

The Chinese rare minnow (*Gobiocypris rarus*), a small cyprinid species endemic to China, is distinguished by its continuous reproductive cycle, short period to sexual maturity, multiple spawning events, and ability to control embryonic development [1]. Due to these unique traits, the Chinese rare minnow is considered the most suitable fish species for experimental research in China [2,3]. However, its restricted distribution range, low natural abundance, and susceptibility to fluctuating environmental conditions have led to its classification as an endangered species in the wild [4,5,6]. In order to facilitate the development of new strains and conserve the genetic resources of the Chinese rare minnow, it is imperative to conduct research on the cryopreservation of its sperm.

Previous research has determined that the quality of cryopreserved sperm from Chinese rare minnows is not adequate [7]. Key determinants of sperm quality include sperm plasma membrane integrity, mitochondrial activity, and DNA integrity [8,9,10]. The plasma membrane plays a crucial role in sperm survival during the fertilization process [11]. Mitochondria are integral to various physiological processes such as capacitation and acrosome reaction. Decreased sperm motility has been associated with mitochondrial impairment and disturbances in metabolic pathways [12,13]. The available evidence indicates that DNA damage is a common occurrence during the cryopreservation of fish sperm [14], which may have implications for the development of offspring [15,16]. Cryoprotectants have been shown to mitigate irreversible mechanical damage resulting from low temperatures during the cryopreservation process, thereby enhancing the overall quality of cryopreserved sperm [10]. Therefore, as already demonstrated in other studies present in the literature on different fish species, we wanted to evaluate the improvement determined by AFPs on the quality of cryopreserved sperm in the Chinese rare minnow.

Antifreeze proteins (AFPs) are a class of proteins that enhance an organism’s ability to withstand freezing temperatures by inhibiting the formation of ice crystals [17]. These proteins are found across a diverse range of organisms, such as animals, plants, bacteria, and fungi. Among AFPs, fish AFPs have been the subject of extensive research and were the earliest identified [18]. The main types of fish AFPs include AFP I, AFP II, AFP III, AFP IV, and antifreeze glycoprotein [19]. While all AFPs exhibit antifreeze properties, they differ in their composition, structure, and interactions with ice crystal surfaces. AFPs have been demonstrated to offer effective protection during the cryopreservation of fish semen [20]. Previous studies have shown that type I and type III AFPs in cryomedium can improve the quality of cryopreserved sperm in common carp (*Cyprinus carpio*) [21], sterlet (*Acipenser ruthenus*) [22,23], and gilthead seabream (*Sparus aurata*) [24]. Therefore, investigating the effects of AFPs on the cryopreservation of Chinese rare minnow sperm is essential.

In this study, we aimed to investigate the protective effect of antifreeze proteins (AFP I and AFP III) at varying concentrations (0.1, 1, 10, and 100 μg/mL) on the cryopreservation of Chinese rare minnow sperm. The optimized technology of ultra-low-temperature sperm cryopreservation for Chinese rare minnow will facilitate the cultivation of new strains and the preservation of germplasm resources.

## 2. Results

### 2.1. Progressive Motility and Kinetic Parameters of Sperm

**Figure 1:**
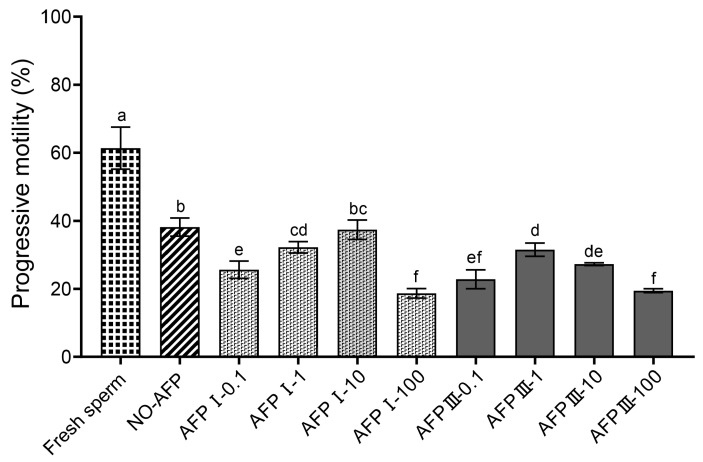
Progressive motility of fresh and cryopreserved sperm of the Chinese rare minnow without/with antifreeze proteins at different concentrations. Different letters above the error bar represent statistical differences (*p* < 0.05).

The progressive motility of fresh sperm was 61.37 ± 6.16%, while the progressive motility of cryopreserved sperm in experimental groups, with and without AFPs, was significantly lower than that of fresh sperm. Among the experimental groups, the cryomedium with 10 μg/mL AFP I exhibited the highest progressive motility (37.40 ± 2.86%). However, there was no significant difference in progressive motility compared to cryopreserved sperm in the group without AFPs (38.17 ± 2.65%) (Figure 1).

**Figure 2:**
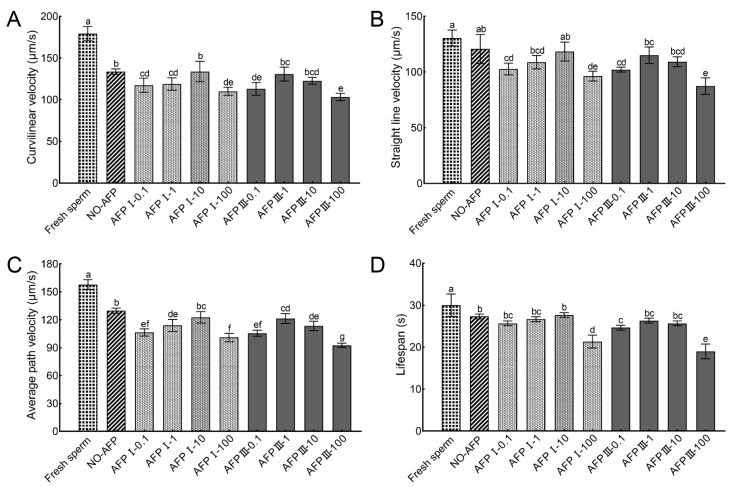
Kinetic parameters of fresh and cryopreserved sperm from Chinese rare minnow without/with antifreeze proteins at different concentrations. (**A**) curvilinear velocity; (**B**) straight-line velocity; (**C**) average path velocity; (**D**) lifespan. Different letters above the error bar represent statistical differences (*p* < 0.05).

The VCL, VSL, VAP, and lifespans of fresh sperm were 179.12 ± 8.44 µm/s, 130.45 ± 7.13 µm/s, 157.67 ± 5.23 µm/s, and 30.00 ± 2.65 s, respectively. The VCL and VAP of cryopreserved sperm in both experimental groups with and without AFPs were significantly lower than those of fresh sperm. Among the experimental groups, cryopreserved sperm supplemented with 10 μg/mL AFP I exhibited the highest values for VCL, VSL, VAP, and lifespan, measuring at 133.77 ± 12.14 µm/s, 118.34 ± 8.55 µm/s, 122.54 ± 6.21 µm/s, and 27.67 ± 0.58 s, respectively. However, no statistically significant differences were observed when compared to the VCL, VSL, VAP, and lifespan of cryopreserved sperm in the group without AFPs (133.66 ± 3.19 µm/s, 120.69 ± 13.09 µm/s, 129.77 ± 2.82 µm/s, and 27.33 ± 0.58 s) (Figure 2).

### 2.2. Sperm Plasma Membrane Integrity

**Figure 3:**
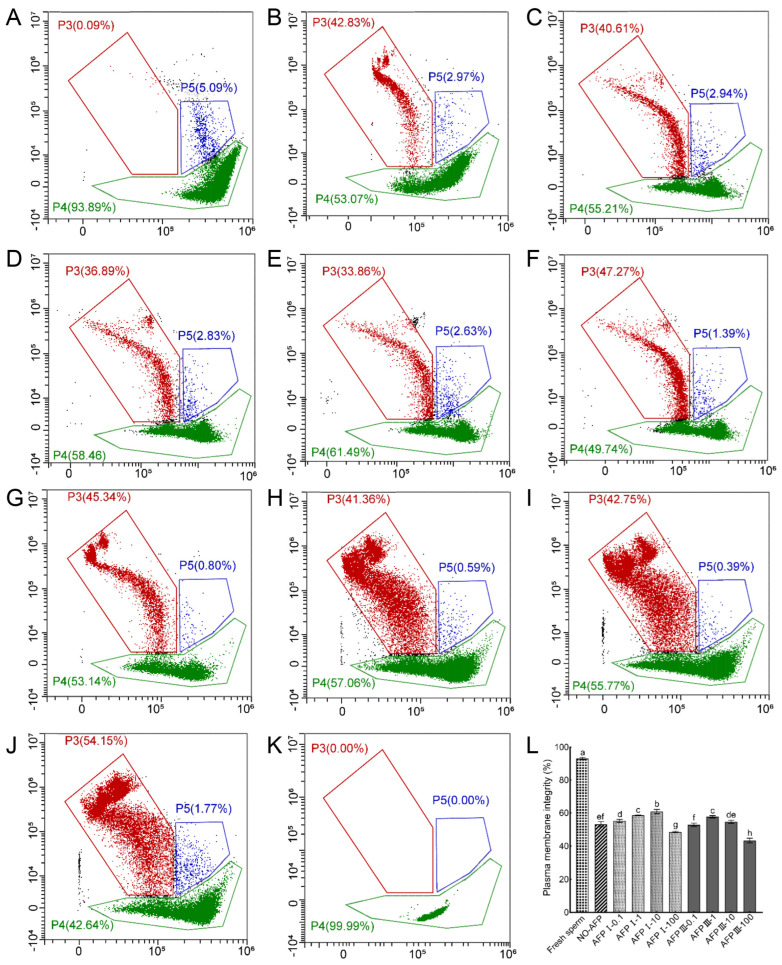
Plasma membrane integrity of fresh and cryopreserved sperm from Chinese rare minnow without/with antifreeze proteins. (**A**–**K**), cytograms for spermatozoa stained with SYBR-14/propidium iodide (PI). P3, sperm with damaged plasma membranes that are dead (SYBR-14^−^/PI^+^); P4, sperm with intact plasma membranes (SYBR-14^+^/PI^−^); P5, sperm in a transitional state that is going to die (SYBR-14^+^/PI^+^). (**A**) fresh sperm; (**B**) cryopreserved sperm without antifreeze proteins; (**C**) cryopreserved sperm with 0.1 μg/mL AFP I; (**D**) cryopreserved sperm with 1 μg/mL AFP I; (**E**) cryopreserved sperm with 10 μg/mL AFP I; (**F**) cryopreserved sperm with 100 μg/mL AFP I; (**G**) cryopreserved sperm with 0.1 μg/mL AFP III; (**H**) cryopreserved sperm with 1 μg/mL AFP III; (**I**) cryopreserved sperm with 10 μg/mL AFP III; (**J**) cryopreserved sperm with 100 μg/mL AFP III; (**K**) negative control; (**L**) histogram of plasma membrane integrity of fresh and cryopreserved sperm from Chinese rare minnow. Different letters above the error bar represent statistical differences (*p* < 0.05).

The assessment of the sperm plasma membrane integrity of the Chinese rare minnow was conducted using a combination of SYBR-14 and PI dyes. Results demonstrated that the plasma membrane integrity of fresh sperm was 93.06 ± 0.76%. Cryopreserved sperm in both experimental groups, with and without antifreeze proteins (AFPs), exhibited significantly lower plasma membrane integrity compared to fresh sperm. Among the experimental groups, the group with 10 μg/mL AFP I added to the cryoprotectants displayed the highest plasma membrane integrity at 60.82 ± 1.27%, which was notably higher than the group without AFPs at 53.12 ± 1.58% (Figure 3).

### 2.3. Mitochondrial Activity

**Figure 4:**
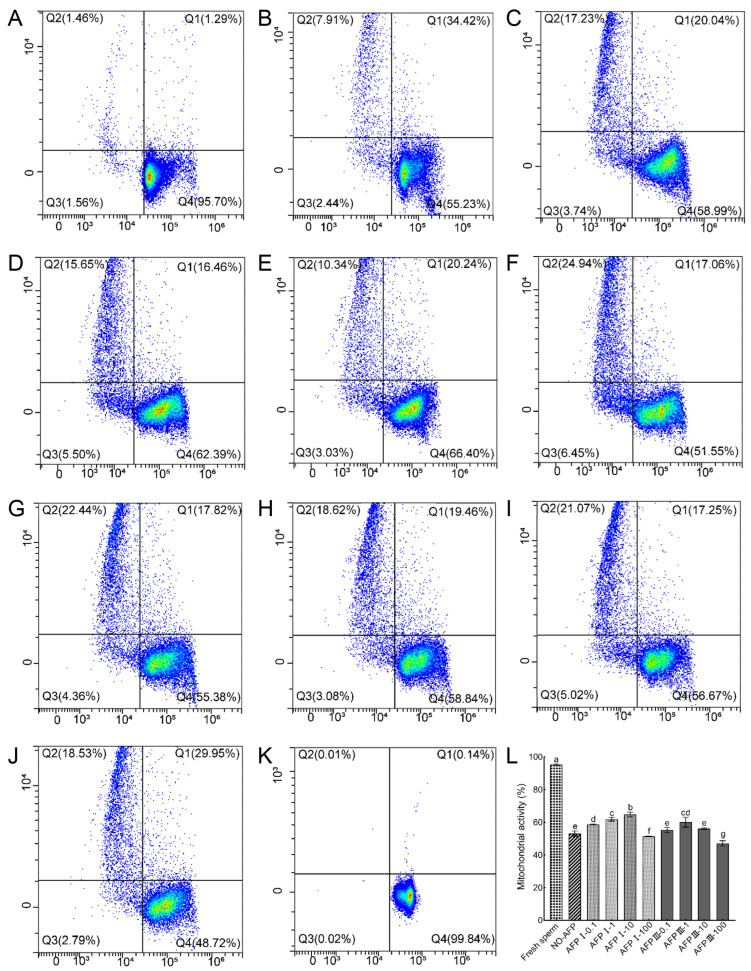
Mitochondrial activity of fresh and cryopreserved sperm from Chinese rare minnow detected by flow cytometer. (**A**–**K**) Cytograms for spermatozoa stained with Rhodamine 123 (Rh123)/propidium iodide (PI). Q1, sperm in a transitional state that is going to die but with active mitochondria (Rh123^+^/PI^+^); Q2, sperm with inactive mitochondria (Rh123^−^/PI^+^); Q3, sperm without mitochondrial transmembrane potential (Rh123^−^/PI^−^); Q4, live sperm with normal mitochondrial function (Rh123^+^/PI^−^). (**A**) fresh sperm; (**B**) cryopreserved sperm without antifreeze proteins; (**C**) cryopreserved sperm with 0.1 μg/mL AFP I; (**D**) cryopreserved sperm with 1 μg/mL AFP I; (**E**) cryopreserved sperm with 10 μg/mL AFP I; (**F**) cryopreserved sperm with 100 μg/mL AFP I; (**G**) cryopreserved sperm with 0.1 μg/mL AFP III; (**H**) cryopreserved sperm with 1 μg/mL AFP III; (**I**) cryopreserved sperm with 10 μg/mL AFP III; (**J**) cryopreserved sperm with 100 μg/mL AFP III; (**K**) negative control; (**L**) histogram of mitochondrial activity of fresh and cryopreserved sperm from Chinese rare minnow. Different letters above the error bar represent statistical differences (*p* < 0.05).

Sperm mitochondrial activity was measured using Rh123 and PI co-staining. The mitochondrial activity of fresh sperm was 95.34 ± 0.35%. The mitochondrial activity of cryopreserved sperm in both experimental groups with and without antifreeze proteins was markedly lower than that of fresh sperm. Among the cryopreserved sperm, the highest mitochondrial activity (64.75 ± 1.43%) was observed in the group with 10 μg/mL AFP I added to the cryoprotectants, significantly higher than that of the group without AFPs (54.08 ± 1.05%) (Figure 4).

### 2.4. DNA Integrity

**Figure 5:**
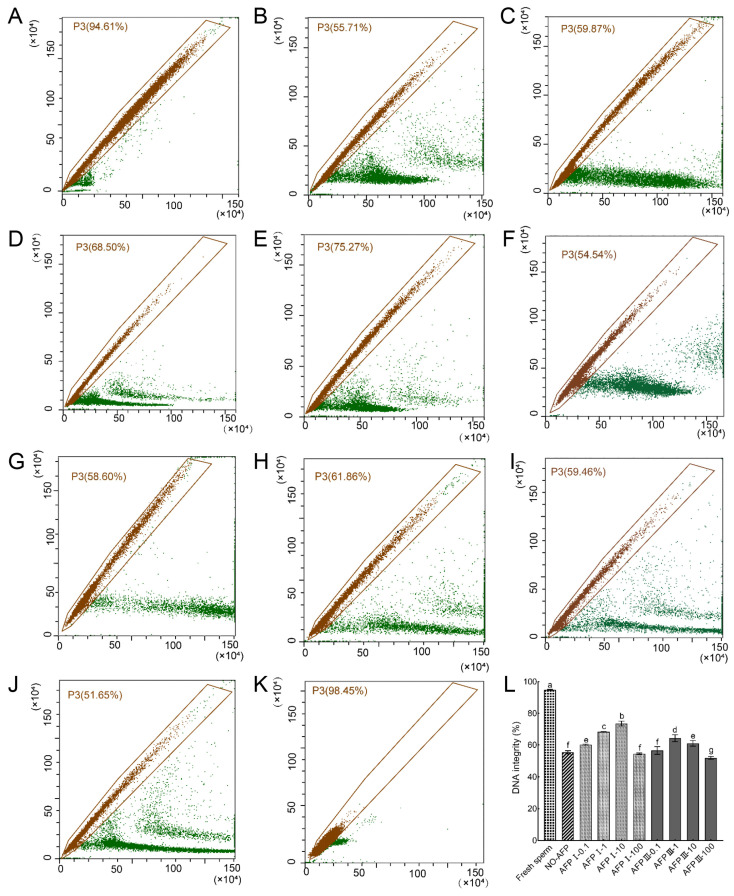
DNA integrity of fresh and cryopreserved sperm from Chinese rare minnow detected by flow cytometer. (**A**–**K**) cytograms for spermatozoa stained with acridine orange (AO). P3, sperm with intact DNA. (**A**) fresh sperm; (**B**) cryopreserved sperm without antifreeze proteins; (**C**) cryopreserved sperm with 0.1 μg/mL AFP I; (**D**) cryopreserved sperm with 1 μg/mL AFP I; (**E**) cryopreserved sperm with 10 μg/mL AFP I; (**F**) cryopreserved sperm with 100 μg/mL AFP I; (**G**) cryopreserved sperm with 0.1 μg/mL AFP III; (**H**) cryopreserved sperm with 1 μg/mL AFP III; (**I**) cryopreserved sperm with 10 μg/mL AFP III; (**J**) cryopreserved sperm with 100 μg/mL AFP III; (**K**) negative control; (**L**) histogram of DNA integrity of fresh and cryopreserved sperm from Chinese rare minnow. Different letters above the error bar represent statistical differences (*p* < 0.05).

Following staining with AO fluorescent dye, the DNA integrity of Chinese rare minnow sperm was assessed. The DNA integrity of fresh sperm was determined to be 94.81 ± 0.34%. In comparison, the DNA integrity of cryopreserved sperm in both experimental groups, with and without AFPs, exhibited a significant decrease when compared to fresh sperm. Specifically, the group supplemented with 10 μg/mL AFP I in the cryoprotectants demonstrated the highest DNA integrity at 73.50 ± 1.53%, which was notably higher than the group without AFPs at 55.38 ± 1.01% (Figure 5).

### 2.5. Fertilization Rate

**Figure 6:**
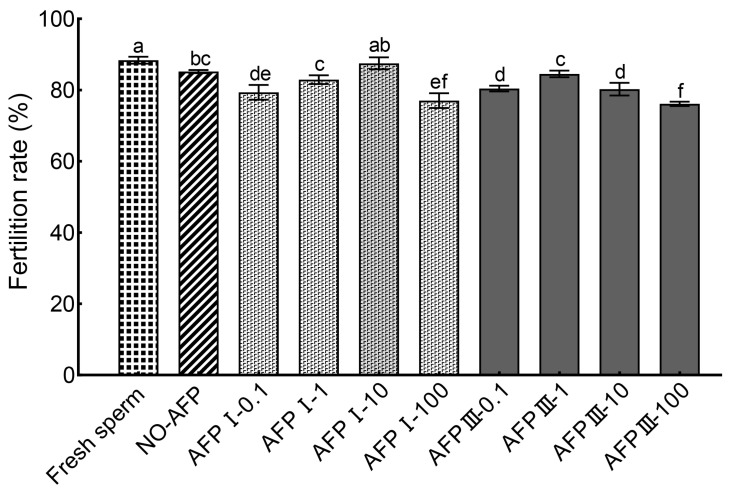
Comparison of fertilization rates between fresh and cryopreserved sperm of Chinese rare minnow. Different letters above the error bar represent statistical differences (*p* < 0.05).

The cryopreserved Chinese rare minnow sperm demonstrated a significantly higher fertilization rate of 87.50 ± 1.68% when cryopreserved in a medium supplemented with 10 μg/mL AFP I compared to other concentrations of AFPs (Figure 6). Furthermore, this highest fertilization rate was not significantly different from the rates observed in the experimental group without AFPs (85.19 ± 0.46%) and fresh sperm (88.37 ± 0.97%). However, the fertilization rate of cryopreserved sperm without AFPs was notably lower than that of fresh sperm.

## 3. Discussion

In the present study, the cryoprotective effects of different concentrations of AFP I and AFP III on Chinese rare minnow sperm were compared. The addition of AFPs to the cryomedium did not result in improved progressive motility and kinetic parameters of the cryopreserved Chinese rare minnow sperm. However, the integrity of the sperm plasma membrane, mitochondrial activity, and DNA integrity were notably enhanced with the inclusion of 10 μg/mL of AFP I in the cryoprotectants. Moreover, there were no significant differences noted in the fertilization rate between fresh sperm and sperm cryopreserved with 10 μg/mL AFP I. The development of an optimized cryomedium could be beneficial for the cultivation of new strains and the preservation of Chinese rare minnow germplasm resources.

AFPs, serving as natural inhibitors of ice crystal formation, have been shown to enhance sperm motility and kinetic parameters by protecting cells and maintaining cell plasma membrane integrity [20]. The impact of AFPs on sperm motility parameters is contingent upon the physiological attributes of distinct fish species and the unique properties of AFPs. Optimal levels of AFPs have the potential to enhance sperm motility and kinetic parameters in certain fish species. The addition of 1 μg/mL AFP III in the cryomedium improved the motility and kinetic parameters of cryopreserved sperm in gilthead seabream [24] and common carp [21]. In contrast, Xin et al. (2018) found no statistically significant disparities in the curvilinear velocity of spermatozoa between fresh and cryopreserved sterlet sperm, regardless of the presence or absence of AFP I or AFPIII supplementation [22]. It appeared that the effects of antifreeze protein on Persian sturgeon (*Acipenser persicus*) sperm during cryopreservation were highly concentration-dependent [25]. Our results were consistent with the opinion of concentrate-dependent action of AFPs. In detail, the progressive motility and kinetic parameters of post-thawed Chinese rare minnow sperm exhibited an increasing trend as the concentration of AFPs increased from 0.1 to 10 μg/mL; however, these values declined when the concentration of AFPs reached 100 μg/mL. The maximum values of progressive motility and kinetic parameters in cryopreserved Chinese rare minnow sperm were achieved at a concentration of 10 μg/mL AFP I, with no statistically significant difference observed compared to the absence of AFPs in the cryomedium. Thus, the impact of AFPs on progressive motility and kinetic parameters of cryopreserved sperm is species-dependent, and the optimal dose does not exceed 10 μg/mL when supplemented in the cryomedium for fish sperm cryopreservation.

An important factor in evaluating cryopreserved sperm is the structural and functional integrity of the membrane [10]. The addition of AFP III in the cryomedium has been shown to significantly improve the plasma membrane integrity of cryopreserved sperm in a variety of species, such as Pacific abalone (*Haliotis discus hannai*) [26], sterlet [27], and gilthead seabream [24]. Moreover, mitochondrial function plays a crucial role in sperm fertilization potential [28]. The freezing, cooling, and thawing procedures have been found to have detrimental effects on sperm mitochondria, leading to decreased adenosine triphosphate (ATP) generation, impaired mitochondrial function, and reduced overall activity [29]. Incorporating 0.1 μg/mL AFP III into the cryomedium for Pacific abalone sperm has been demonstrated to enhance mitochondrial function in cryopreserved sperm [26]. Additionally, the integrity of sperm DNA serves as a crucial factor in assessing sperm quality [30]. In the present study, the incorporation of AFP I at concentrations below 100 μg/mL in the cryomedium significantly improved the integrity of the plasma membrane, mitochondrial activity, and DNA integrity of cryopreserved sperm in Chinese rare minnow. The protective mechanism of AFPs is attributed to their interaction with sperm membrane constituents, particularly lipid organization [18]. Throughout the cryopreservation process, there is an increase in sperm lipid peroxidation which subsequently leads to heightened production of reactive oxygen species (ROS), resulting in diminished sperm mitochondrial activity and structural damage to the sperm membrane [24]. The incorporation of AFPs during sperm cryopreservation has been shown to mitigate ROS production, thereby stabilizing the sperm membrane structure and reducing sperm DNA damage [30,31]. Further research is needed to investigate the impact of ROS on the quality of cryopreserved Chinese rare minnow sperm.

The fertilization rate serves as a critical parameter for assessing the quality of cryopreserved sperm. The addition of AFP III to the extender improved the fertilization rate of cryopreserved sperm in gilthead seabream [24], making no significant difference with that of fresh sperm. However, the supplement AFPs in the cryomedium did not enhance the fertilization rate in sterlet [23]. In the present study, it was observed that the fertilization rate of cryopreserved sperm from Chinese rare minnows was significantly higher when cryopreserved in a cryomedium supplemented with 10 μg/mL AFP I compared to other concentrations of AFPs. Furthermore, although the progressive motility and kinetic parameters of cryopreserved sperm with 10 μg/mL AFP I were comparable to or lower than those of cryopreserved sperm without AFPs, the fertilization rate of the former was higher. Notably, the fertilization rate of the cryopreserved sperm with 10 μg/mL AFP I showed no significant difference when compared to fresh sperm. The results of the present study indicate that the plasma membrane integrity, mitochondrial activity, and DNA integrity of cryopreserved Chinese rare minnow sperm with 10 μg/mL AFP I were notably higher compared to cryopreserved sperm without AFPs, implying a potential correlation with fertilization rates in Chinese rare minnow sperm. Further investigation is required to elucidate this relationship in future research.

## 4. Materials and Methods

### 4.1. Sample Collection

The Chinese rare minnow specimens utilized in this study were obtained from artificially domesticated male broodstock at the Institute of Hydrobiology, Chinese Academy of Sciences (Wuhan, China). The total length and body weight of mature males were 5.59 ± 0.28 cm and 1.56 ± 0.25 g, respectively. Sperm samples, each with an approximate volume of 10 µL, were individually collected through abdominal stripping followed by capillary aspiration. These samples were temporarily stored in 0.2 mL Eppendorf (EP) tubes (Axygen Biotechnology Co., Ltd., Hangzhou, China) placed in an ice box. Throughout the collection process, precautions were taken to shield the stripped sperm from direct light exposure and contamination by blood, urine, and feces. The sampling procedure followed the animal welfare guidelines and relevant regulations of the Institute of Hydrobiology, Chinese Academy of Sciences, and the Yangtze River Fisheries Research Institute, Chinese Academy of Fishery Sciences.

### 4.2. Sperm Progressive Motility and Kinetic Parameters Assessment

The CEROS II computer-assisted sperm analysis system (Hamilton Thorne, Beverly, MA, USA) was utilized to measure lifespan (defined as the time from activation by ultrapure water until 90% of the sperm exhibited tremor-like movement) and various motility parameters, including curvilinear velocity (VCL, µm/s), straight-line velocity (VSL, µm/s), and average path velocity (VAP, µm/s). Meanwhile, the progressive motility of the sperm was determined. Semen samples with motility exceeding 90% were utilized for subsequent sperm cryopreservation experiments, with each experiment repeated at least three times.

### 4.3. Sperm Cryopreservation

The cryoprotectant utilized for Chinese rare minnow sperm consisted of 8% methanol (Sinopharm Chemical Reagent Co., Ltd., Shanghai, China) and 90 mmol/L glucose (Amresco, Radnor, PA, USA) in buffered sperm motility-inhibiting solution (BSMIS) as previously described [7] and stored in ice after it was prepared. Subsequently, varying concentrations (0.1, 1, 10, and 100 μg/mL) of AFP I and AFP III (A/F Protein Inc., Waltham, MA, USA) were incorporated into the cryoprotectant mixture [23]. The sperm samples were then diluted and combined with the cryoprotectants at a ratio of 1:5 before being loaded into 0.25 mL straws (Fujihira industry Co., Ltd., Tokyo, Japan). The final concentration of sperm in each straw was about 1 × 10^9^ sperm/mL. The straws were equilibrated at 0 °C for 5 min, positioned 3 cm above liquid nitrogen for 10 min, and subsequently stored in liquid nitrogen. After a 24-h period, the sperm samples were thawed by immersing the straws in a water bath at 40 °C for 5–8 s. A drop of 15 µL distilled water was added to the slide, and subsequently, 0.3 μL sperm were dipped for activation. Immediately, the computer-assisted sperm analysis system was used to record sperm progressive motility, kinetic parameters, and lifespan.

### 4.4. Sperm Plasma Membrane Integrity Evaluation

Sperm plasma membrane integrity was evaluated using the LIVE/DEAD Sperm Viability Kit (Thermo Fisher Scientific Inc., Waltham, MA, USA). The semen sample was diluted with phosphate-buffered saline (PBS) to a concentration of 3 × 10^7^ sperm/mL. Subsequently, SYBR-14 dye (final concentration of 100 nmol/L) and propidium iodide (PI, final concentration of 12 µmol/L) were added to the diluted sample, which was then mixed and incubated for 10 min at 37 °C in the absence of light. The sample was subjected to two wash cycles with PBS and centrifuged at 600× *g* for 5 min. The sperm pellet was then resuspended in fresh PBS. Flow cytometer (Beckman Coulter, Inc., Brea, CA, USA) analysis was performed to measure the fluorescence signals emitted by SYBR-14 and PI within sperm, with a total of 20,000 sperm analyzed per sample.

### 4.5. Mitochondrial Activity Evaluation

The semen sample was diluted with PBS to a concentration of 3 × 10^7^ sperm/mL. Rhodamine 123 (Rh123) at a final concentration of 10 µg/mL (Sigma-Aldrich, St. Louis, MO, USA) and PI were added to the diluted sample, and then incubated for 10 min at 37 °C in the absence of light. The sample underwent two wash cycles with PBS, followed by centrifugation at 600× *g* for 5 min. The sperm pellet was subsequently resuspended in fresh PBS. Flow cytometry analysis was conducted to quantify the fluorescence signals emitted by Rh123 and PI within the sperm, with 20,000 sperm cells analyzed per sample.

### 4.6. DNA Integrity Evaluation

The semen sample was diluted with PBS to a concentration of 3 × 10^7^ sperm/mL. Following dilution, acridine orange (AO) at a final concentration of 100 µg/mL (Sigma-Aldrich) was added to the sample, which was then incubated for 10 min at 37 °C in the absence of light. Subsequently, the sample was washed two times with PBS and centrifuged at 600× *g* for 5 min. The resulting sperm pellet was resuspended in PBS. Flow cytometry analysis was performed to quantify the fluorescence signals emitted by AO within the sperm, with a total of 20,000 sperm analyzed for each sample.

### 4.7. Fertilization Test

Following a 24 h period of sperm cryopreservation, the quality of the sperm upon thawing was evaluated, and subsequent fertilization experiments were carried out. Adult female fish were carefully selected, and their eggs were obtained through gentle abdominal pressure, with each female yielding between 100 and 300 eggs. The collected eggs from multiple females were combined and distributed evenly into culture dishes, each containing 50 to 70 eggs. The ratio of sperm to eggs utilized was one million sperm to one egg. Thawed semen was introduced to the culture dishes and promptly mixed with water for 30 s. After a 2 min incubation period, the mixture was rinsed twice with water and placed in an incubator set at 25 °C. Upon reaching the gastrula stage, the fertilization rate was determined by the formula: fertilization rate = number of embryos at the gastrula stage/total number of eggs × 100.

### 4.8. Statistical Analysis

The data were presented as mean ± standard deviation (mean ± SD) and analyzed using SPSS 23.0 software (SPSS Inc., Chicago, IL, USA). The significance of difference in sperm motility between groups was assessed by one-way analysis of variance (ANOVA). Prior to conducting ANOVA, the normality and homogeneity of variances were determined by the Shapiro–Wilk and Bartlett’s tests, respectively. Subsequent multiple comparisons were conducted employing Duncan’s method, with a significance level of *p* < 0.05 denoting a statistically significant distinction. The results were plotted using GraphPad Prism 9.5 software (GraphPad Software, Inc., San Diego, CA, USA).

## 5. Conclusions

This study found that the addition of 10 μg/mL AFP I to the cryomedium for Chinese rare minnow sperm did not result in significant improvements in sperm progressive motility and kinetic parameters. However, it did significantly enhance sperm plasma membrane integrity, mitochondrial activity, and DNA integrity. Furthermore, there were no notable differences observed in the fertilization rate between fresh sperm and cryopreserved sperm supplemented with 10 μg/mL AFP I. These results indicate a successful improvement in the quality of cryopreserved Chinese rare minnow sperm, supporting the potential for cultivating new strains and preserving germplasm resources for this species.

## Data Availability

All data in this study are presented in this published article.

## References

[1] Wang J.W. (1992). Reproductive biology of *Gobiocypris rarus*. Acta Hydrobiol. Sin..

[2] Zhong X., Xu Y., Liang Y., Liao T., Wang J. (2005). The Chinese rare minnow (*Gobiocypris rarus*) as an in vivo model for endocrine disruption in freshwater teleosts: A full life-cycle test with diethylstilbestrol. Aquat. Toxicol..

[3] Wang J., Cao W. (2017). *Gobiocypris rarus* as a Chinese native model organism: History and current situation. Asian J. Ecotoxicol..

[4] He Y., Wang J., Blanchet S., Lek S. (2012). Genetic structure of an endangered endemic fish (*Gobiocypris rarus*) in the upper Yangtze River. Biochem. Syst. Ecol..

[5] Xiong D., Xie C., Xia L. (2009). Threatened fishes of the world: *Gobiocypris rarus* Ye and Fu, 1983 (Cypinidae). Environ. Biol. Fishes.

[6] Zhang E., Chen X.Y. *Gobiocypris rarus*. The IUCN Red List of Threatened Species 2023: E.T212878417A212878419.

[7] Li X., Ye H., He Y.F., Yu L., Cheng P.L., Du H., Wu J.M. (2024). Sperm cryopreservation of *Gobiocypris rarus*. Acta Hydrobiol. Sin..

[8] Cabrita E., Sarasquete C., Martínez-Páramo S., Robles V., Beirão J., Pérez-Cerezales S., Herráez M.P. (2010). Cryopreservation of fish sperm: Applications and perspectives. J. Appl. Ichthyol..

[9] Kowalski R.K., Cejko B.I. (2019). Sperm quality in fish: Determinants and affecting factors. Theriogenology.

[10] Xin M., Niksirat H., Shaliutina-Kolešová A., Siddique M.A.M., Sterba J., Boryshpolets S., Linhart O. (2020). Molecular and subcellular cryoinjury of fish spermatozoa and approaches to improve cryopreservation. Rev. Aquac..

[11] Cabrita E., Martínez-Páramo S., Gavaia P.J., Riesco M.F., Valcarce D.G., Sarasquete C., Herráez M.P., Robles V. (2014). Factors enhancing fish sperm quality and emerging tools for sperm analysis. Aquaculture.

[12] Alavi S.H., Cosson J., Bondarenko O., Linhart O. (2019). Sperm motility in fishes: (III) diversity of regulatory signals from membrane to the axoneme. Theriogenology.

[13] Dzyuba V., Cosson J. (2014). Motility of fish spermatozoa: From external signaling to flagella response. Reprod. Biol..

[14] Jeuthe H., Palaiokostas C., Johannisson A. (2022). DNA fragmentation and membrane integrity in sperm of farmed Arctic charr (*Salvelinus alpinus*). Aquaculture.

[15] Sandoval-Vargas L., Silva Jimenez M., Risopatron Gonzalez J., Villalobos E.F., Cabrita E., Valdebenito Isler I. (2021). Oxidative stress and use of antioxidants in fish semen cryopreservation. Rev. Aquacult..

[16] Pérez-Cerezales S., Martínez-Páramo S., Beirão J., Herráez M.P. (2010). Fertilization capacity with rainbow trout DNA-damaged sperm and embryo developmental success. Reproduction.

[17] Bianco V., Espinosa J.R., Vega C. (2020). Antifreeze proteins and homogeneous nucleation: On the physical determinants impeding ice crystal growth. J. Chem. Phys..

[18] Xiang H., Yang X., Ke L., Hu Y. (2020). The properties, biotechnologies, and applications of antifreeze proteins. Int. J. Biol. Macromol..

[19] Baskaran A., Kaari M., Venugopal G., Manikkam R., Joseph J., Bhaskar P.V. (2021). Antifreeze proteins (Afp): Properties, sources and applications—A review. Int. J. Biol. Macromol..

[20] Abed-Elmdoust A., Uysal O., Rahimi R., Farahmand Y. (2021). Antifreeze proteins are robust cryoprotectants for sperm cryopreservation in fishes: A systematic review and meta-analysis. Aquaculture.

[21] Shaliutina-Kolešová A., Dietrich M., Xian M., Nian R. (2019). Seminal plasma transferrin effects on cryopreserved common carp *Cyprinus carpio* sperm and comparison with bovine serum albumin and antifreeze proteins. Anim. Reprod. Sci..

[22] Xin M., Sterba J., Shaliutina-Kolesova A., Dzyuba B., Lieskovska J., Boryshpolets S., Siddique M.A.M., Kholodnyy V., Lebeda I., Linhart O. (2018). Protective role of antifreeze proteins on sterlet (*Acipenser ruthenus*) sperm during cryopreservation. Fish Physiol. Biochem..

[23] Xin M., Tučková V., Rodina M., Kholodnyy V., Dadras H., Boryshpolets S., Shaliutina-Kolešová A., Linhart O. (2018). Effects of antifreeze proteins on cryopreserved sterlet (*Acipenser ruthenus*) sperm motility variables and fertilization capacity. Anim. Reprod. Sci..

[24] Beirão J., Zilli L., Vilella S., Cabrita E., Schiavone R., Herráez M.P. (2012). Improving sperm cryopreservation with antifreeze proteins: Effect on gilthead seabream (*Sparus aurata*) plasma membrane lipids. Biol. Reprod..

[25] Abed-Elmdoust A., Farahmand H., Mojazi-Amiri B., Rafiee G., Rahimi R. (2015). Novel droplet vitrification combined with fish antifreeze protein type III enhances cryoprotection of semen in wild endangered Persian sturgeon *Acipenser persicus* (Borodin, 1897). Aquac. Res..

[26] Hossen S., Sharker M.R., Cho Y., Sukhan Z.P., Kho K.H. (2021). Effects of antifreeze protein III on sperm cryopreservation of Pacific abalone, *Haliotis discus hannai*. Int. J. Mol. Sci..

[27] Dadras H., Golpour A., Rahi D., Lieskovská J., Dzyuba V., Gazo I., Policar T. (2022). Cryopreservation of sterlet, *Acipenser ruthenus* spermatozoa: Evaluation of quality parameters and fine ultrastructure. Front. Mar. Sci..

[28] Ulloa-Rodríguez P., Figueroa E., Díaz R., Lee-Estevez M., Short S., Farías J.G. (2017). Mitochondria in teleost spermatozoa. Mitochondrion.

[29] Figueroa E., Valdebenito I., Zepeda A.B., Figueroa C.A., Dumorné K., Castillo R.L., Farias J.G. (2017). Effects of cryopreservation on mitochondria of fish spermatozoa. Rev. Aquac..

[30] Shaliutina-Loginova A., Loginov D.S. (2023). Oxidative stress and DNA fragmentation in frozen/thawed common carp *Cyprinus carpio* sperm with and without supplemental proteins. Anim. Reprod. Sci..

[31] Ekpo M.D., Xie J., Hu Y., Liu X., Liu F., Xiang J., Zhao R., Wang B., Tan S. (2022). Antifreeze proteins: Novel applications and navigation towards their clinical application in cryobanking. Int. J. Mol. Sci..

